# Special Issue “II-VI Semiconductor Nanocrystals and Hybrid Polymer–Nanocrystal Systems”

**DOI:** 10.3390/nano11020467

**Published:** 2021-02-12

**Authors:** Marco Anni

**Affiliations:** Dipartimento di Matematica e Fisica “Ennio De Giorgi”, Università del Salento, Via per Arnesano, 73100 Lecce, Italy; marco.anni@unisalento.it

The continuous need to improve the performance of photonic, electronic and optoelectronic devices has stimulated research toward the development of innovative semiconducting materials which display better properties with respect to standard bulk semiconductors.

In this context, organic conjugated molecules and colloidal semiconductor nanocrystals (NCs) have received considerable attention in the last few decades.

Conjugated molecules are able to combine the easy processability and the chemical flexibility of plastic materials with the active properties of semiconductors, such as charge mobility, electroluminescence and optical gain.

These properties present opportunities for the development of engineered molecules with widely tunable active properties and deposited by simple techniques, to be exploited as active materials in field effect transistors, light emitting diodes, solar cells and lasers.

Concerning II-VI NCs, the development of colloidal synthesis processes in solutions allowed the realization of a wide range of different NCs, with different chemical compositions, shapes and surface functionalization. Moreover, the possibility of finely controlling the NC’s size below the exciton Bohr radius allows broad emission color tunability to be obtained by controlling the quantum confinement effect.

Rather interestingly, many organic molecules are soluble in common organic solvents, in which NCs can also be dispersed, thus allowing the easy mixing of organic and inorganic active materials in solutions to realize hybrid thin films.

This possibility paves the way towards the realization of hybrid organic NC active films able to combine the best of both worlds. For example, the presence of the organic matrix can allow the improvement of the NC’s films forming properties or the enhancement of their operational stability. In a similar way, the inorganic component of the blend can be exploited to improve the film charge mobility with respect to that of purely organic films.

This Special Issue is focused on the recent research on colloidal II-VI NCs and on hybrid organic NC films, in order to evaluate the ongoing research into these systems.

In their paper, S. H. Kim et al. [1] investigate the role of the nanocrystal size and shape in the optical properties of CdSe quantum dots realized by hot injection. In particular, the authors demonstrate, by high-resolution TEM measurements, that the nanocrystals typically show an elongated shape and, by X-ray diffraction, that the crystal structure depends on the NC’s dimensions. A mixture of wurzite and zinc blende structure is observed in the smaller NCs, while a pure zinc blende structure is present in the larger NCs, also characterized by higher crystallinity. The authors also attribute the electronic transitions related to the first six absorption peaks and demonstrate that the excitonic absorption peaks are increasingly “hidden” with decreasing QD size because of the crystal structure and crystalline quality.

A useful example of the possibility of improving the active properties of NCs by acting on the synthesis process is described by F. Huang et al. [2], who propose sulfur passivation as an effective means of tuning the surface sites of heavy metal-free ZnSe nanorods. These systems have been recently proposed as efficient materials for photoanodes of photoelectrochemical cells (PECs) for water splitting, but the device performance is limited by the charge carrier trapping in defect states on the NC’s surface. The authors demonstrate, by X-ray photoelectron spectroscopy (XPS) analysis, that upon treatment with sodium sulfide, $S_{2}^{-}$ ions substitute a large fraction of surface ligands and combine with the Zn termination atoms to form a ZnS monolayer, thus efficiently passivating the surface states. As a consequence, a photocurrent improvement of approximately 20% is demonstrated in a PEC with a TiO2/ZnSe passivated nanorod photoanode, with respect to a comparable device with non-functionalized rods. An improvement in the stability of the device is also demonstrated, clearly evidencing the potential of proper passivation of the II-VI semiconductor NC’s surface as an efficient approach to address the efficient charge transfer and stability of photoelectrochemical cells based thereon. 

Another example of the possibility to engineer the active properties of NCs is presented by G. Xing et al. [3], who report on the synthesis and characterization of ultrathin ZnS nanowires, with diameters down to 1 nm and lengths up to 330 nm. The authors describe a ligand-induced low-temperature precursor thermolysis route for the nanowire preparation of ultrathin ZnS nanowires, demonstrating excellent control of both the diameter and the length. The investigation of the absorption spectra dependence on the wire geometry evidences efficient absorption in the UV range, with an absorption edge mainly determined by the diameter and not appreciably depending on the length. The combination of very small diameter and long length determines a high surface/volume ratio, which leads to a clearly improved adsorption efficiency with respect to standard spherical nanocrystals. Finally, the authors also demonstrate improved photocatalytic properties by investigating the degradation dynamics of Eosin B, used as a target contaminant.

The Special Issue also includes three papers on hybrid organic NC systems.

The paper by V. Dzhagan et al. [4] investigates in depth the origin of photoluminescence (PL) intensity variation under continuous laser illumination in a polymer–NC film. Several previous experiments reported a wide variety of results on the evolution of PL intensity and line shape over time, indicating that several physical and chemical processes, significantly different between particular studies, take place when NCs are continuously illuminated by laser light. The current experiment substantially improves the understanding of these features thanks to the investigation of several active NCs (CdSe, CdSe/ZnS, CdSe/CdS, CdTe and ZnO) embedded in different host polymers, and to the measurements of the PL dependence on time under continuous illumination at fixed excitation density, temperature at fixed excitation density and on the excitation density at different times. The main evidence of the paper is that photo-brightening or photodegradation takes place, depending on the active NC and the host polymer.

The broadly systematic nature of the experiment allows the authors to conclude that the most probable mechanism behind the photo-enhancement for this type of system is the redistribution of the photoexcited charge carriers over the trap states. In particular, the accumulation of photo-excited charges in trap states, leading to a saturation of the latter, plays a key role in the subsequent enhancement of the radiative recombination.

In their paper, F. Rodríguez-Mas et al. [5] discuss the possible development of hybrid polymer–NC active films for LEDs. In particular, the authors investigate the possibility of mixing in a single layer poly(N-vinylcarbazole) (PVK) and colloidal CdS NCs capped with thiophenol in order to realize hybrid LEDs with engineered electrical properties and tunable electroluminescence spectra. The authors demonstrate that a device exploiting an active layer realized by simple mixing in a solution of PVK and NCs only shows resistive behavior, without any electroluminescence, and ascribe this device failure to the poor solubility of the PVK (toluene) and the NC (DMSO) solvents, resulting in poor film uniformity due to the aggregation of the NCs and the presence of nanometric holes.

A possible solution is developed, based on drying and redispersion of NCs in toluene, thus allowing the realization of hybrid films with improved morphology. The LEDs’ performance is investigated for four different PVK:NC relative content levels, demonstrating a progressive increase in the current density and a decrease in the turn on voltage as the NC content is increased, together with electroluminescence spectrum tuning. The demonstration of electroluminescence from the DMSO-free CdS NCs presents the possibility of fabricating optimized white LEDs with nearly uniform emission intensity in the entire visible range of wavelengths. 

The Special Issue is completed by a review paper by M. Anni [6] about the actual state of the art of the understanding of the photophysics of inert systems made by the combination of organic molecules and semiconductor NCs and of their possible device applications. In particular, the paper describes in depth the main experiments in the investigation of the interaction between the organic and the inorganic components of the hybrid systems, describing several experiments that allowed observation of the presence of exciton transfer and/or charge transfer. Concerning the applicative perspectives, the paper describes the state of the art of optically pumped lasers based both on NCs and on polymer–NC blends. Finally, the state of the art of light emitting diodes based on hybrid active materials is also discussed.
